# Welcome to TheScientificWorld

**DOI:** 10.1100/tsw.2000.1

**Published:** 2001-04-04

**Authors:** Eric Tomlinson

## Abstract

Our business is in providing you with the tools and resources to help you to conduct research better. This includes our meeting planning resource, WorldMeet, and several community channels including NewsLink and NewsLab that allows you to personalize scientific news to reflect your interests. PuPAlert enables us automatically to alert Members to new information based on their stated areas of scientific interest. (“Dont forget CoolStuff!” enthuses Brett). We hope you will come to regard TheScientificWorld as an essential web site that provides you with the information tools and resources you need to conduct research. There will be many enhancements to our own offerings in the months and years to come with a focus on providing community services to enable you to connect to your peers. On behalf of our employees and many partners may I sincerely welcome you to TheScientificWorld, your personalized network of scientific information, research tools and scientific resources.

During the play, Heisenberg and Bohr attempt to recall the dates when they had last attended a conference together. Was it '37 or '35, they asked as they walked together, hidden from possible eavesdroppers. Their scientific doggerel revealed the then elitism of science, fraught with discrimination, and often misunderstood by the public. Science was generally disregarded by government as it placed its attention on theory and invention rather than on innovation. Scientists formed affinities through 'schools of thought'; meetings were few and far between; the written paper, with its laborious preparation and slow dissemination, was the mechanism through which science was defined and communicated. Equipment was often made in situ (Heisenberg lashing up his own cyclotron in a former wine cellar cut into the side of a German mountain tells it all); materials were often poorly characterized; and the knowledge of novel methods was patchy.

How necessarily different it all is today. Scientific exchange is more open, with scientists having constant contact with their peers (via email, videoconferences, international symposia, and the like). Travel is much less expensive and time-consuming. Highly functional equipment, high-quality materials and robust methods and protocols are commercially available. And huge volumes of scientific information are now pouring out of laboratories (governmental, academic and industrial).

Most scientists agree their work is to perform original research to create knowledge and / or to produce innovative products. Many approaches are taken to achieve these goals but to conduct research, scientists have a fundamental need to have easy access to authoritative scientific information and to be able to assess the worth of this information prior to conducting experiments using scientific equipment, materials and unique methods. Allied to this is the increasing need to organize and search the scientific literature. Some estimate that life scientists can spend the majority of their working time searching for scientific information. With the volume of information increasing so rapidly, the time from information creation to publication requires a rethinking of the traditional methods of publishing in printed journals. New electronic methods are being described and information technology techniques enabling rapid identification and extraction of new knowledge from data (e.g., data mining and cluster analysis) are of increasing importance to science. For example, data mining can automate the identification, extraction and normalization of information from unstructured text, such as technical papers or genetic sequence datasets. One application of data extraction is the automatic population of a database of protein function generated from searching the scientific literature. This type of application has been validated at the National Library of Medicine using their text processing capabilities (Metamap). Other advances in the management of biological information include simulation models of cell processes and the linkage of visual and digital images of physiological and genetic processes.

Science exists to understand. Niels Bohr is quoted as saying "We shall never understand anything until we have found some contradictions". TheScientificWorld aims to enhance and accelerate scientific and medical research by integrating the key sources of scientific research information (databases, datasets and single articles) into a coherent and navigable form. Members can use our Knowledge Network for Science to access freely the world's most comprehensive collection of scientific research information that can be purchased on a per article basis, **SciBase**. TheScientificWorld web site will present information in innovative ways including the integration of large datasets with single articles to create specialized ScienceWorlds where information is dynamically presented according to the specific scientific interests of a participating scientist. In addition we have introduced a new innovative electronic forum, ***i*-Publish**, for immediate worldwide publication of high-quality peer-reviewed research. As Bohr stated (at least according to Frayn): "We don't do science for ourselves, we do it to explain science to others"). Work submitted to *i*-Publish will receive prompt review by renowned editors. *i*-Publish allows for multimedia content and dynamic interaction between the content and other data sources.

We hope you will come to regard **TheScientificWorld** as an essential web site that provides you with the information tools and resources you need to conduct research.

There will be many enhancements to our own offerings in the months and years to come with a focus on providing community services to enable you to connect to your peers.

On behalf of our employees and many partners may I sincerely welcome you to TheScientificWorld, your personalized network of scientific information, research tools and scientific resources.

Eric Tomlinson, DSc, PhD
Chief Executive Officer
TheScientificWorld
